# Synthesis of nanostructured powders and thin films of iron sulfide from molecular precursors†

**DOI:** 10.1039/c8ra04917c

**Published:** 2018-08-15

**Authors:** Laila Almanqur, Inigo Vitorica-yrezabal, George Whitehead, David J. Lewis, Paul O'Brien

**Affiliations:** School of Chemistry, University of Manchester Oxford Road Manchester M13 9PL UK paul.o'brien@manchester.ac.uk; School of Materials, University of Manchester Oxford Road Manchester M13 9PL UK david.lewis-4@manchester.ac.uk

## Abstract

Iron(iii) xanthate single-source precursors [Fe(S_2_COR)_3_] (R = methyl, ethyl, isopropyl and 1-propyl) were used to deposit iron sulfide thin films and nanostructures by two simple, efficient and low-cost methods (spin coating and solid state deposition). The single-crystal X-ray structures of the iron(iii) *n*-propyl xanthate and iron(iii) iso-propyl xanthate have been determined. Thermogravimetric analysis (TGA) studies of the complexes shows that decomposition of the complexes produces iron sulfide, pyrite or trolite. The crystallinity of iron sulfide thin films and powder samples was studied using X-ray diffraction (XRD), and their morphology was studied by scanning electron microscopy (SEM).

## Abstract 2

Iron(iii) xanthate single-source precursors were used to deposit iron sulfide thin films and nanostructures by spin coating and solid state deposition.

## Graphical abstract

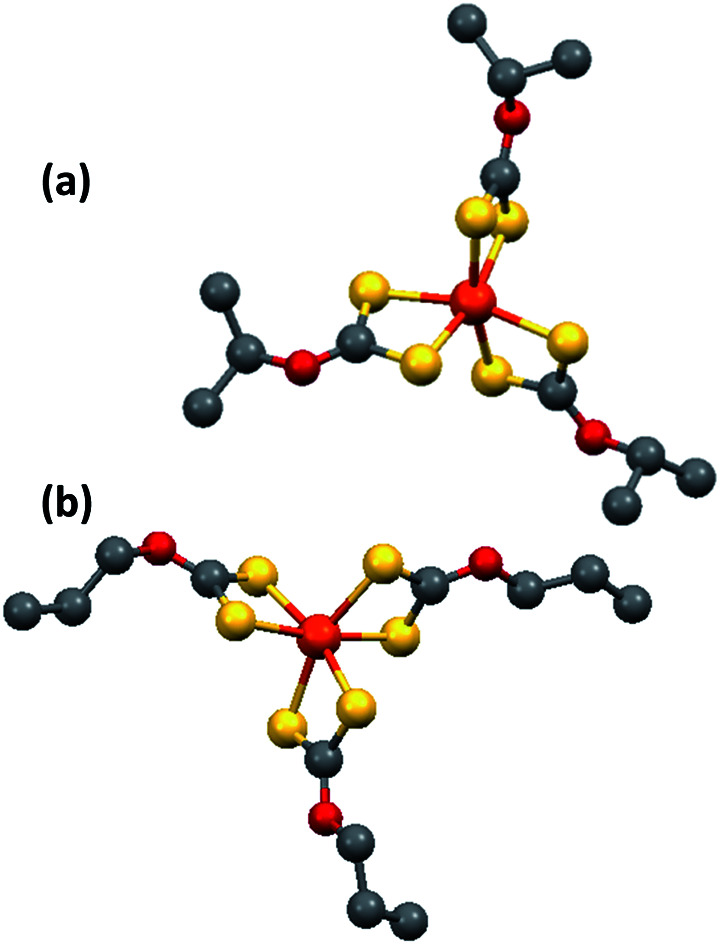

## Introduction

Iron chalcogenides have attracted considerable attention over the last decade, due to their interesting optical, electrical and magnetic properties.^1,2^ Iron sulfide is the most earth-abundant chalcogenide mineral, and is used in several important applications, including batteries, catalytic processes and biomedical appliances and is also a potentially useful material for sustainable and inexpensive photovoltaics applications.^3–5^ It has many potential advantages over other materials, including its low cost, high abundance, negligible toxicity and unique magnetic, electric and optical properties.^6–9^ Iron sulfide crystallises in many different phases; these include pyrite (cubic FeS_2_), marcasite (orthorhombic FeS_2_), mackinawite (Fe_1+*x*_S), troilite (FeS), pyrrhotite (Fe_1−*x*_S), greigite (cubic spinel Fe_3_S_4_) and smythite (Fe_3+*x*_S_4_).^10–12^ The optical, magnetic and electrical properties of iron sulfides vary according to the stoichiometric ratio between the iron and sulfur atoms.^13^ Of these compounds, cubic pyrite (FeS_2_) is of interest for its potential use as an absorber material in photovoltaic and photo-electrochemical applications because of its suitable band gap (0.80–0.95 eV) and high absorption coefficient (∼10^5^ M^−1^ cm^−1^) extending over the visible energy range.^14–18^ The favoured phases for Li-ion batteries are suggested to be troilite (FeS) and pyrrhotite (Fe_1−*x*_S). For supercapacitor applications, troilite (FeS) and greigite (Fe_2_S_3_) are potential candidate materials.^19^

A variety of methods have been used to prepare iron sulfide nanoparticles including hot injection^20–22^ and colloidal methods,^23^ hydrothermal methods,^24^ and solvothermal methods.^25,26^ Nanoscale iron sulfide has been synthesized with various morphologies by the decomposition of several single source precursors such as iron alkyl dithiocarbamates,^27,28^ iron polysulfides, iron thiobiurets^29^ iron diethyldithiophosphates,^30^ FeS cubane clusters,^31^ iron thiosemicarbazones^32^ and iron alkyl xanthates.^33^ In addition, a great deal of attention has been paid to the deposition and characterization of iron sulfide thin films. Several deposition techniques have been employed to deposit iron sulfide thin films, including chemical bath deposition,^34^ chemical vapour deposition,^35^ chemical bath deposition,^36^ metal–organic chemical vapour deposition (MOCVD),^37^ and aerosol-assisted chemical vapour deposition (AACVD).^38–40^

Herein we report the synthesis of tris (*O*-alkylxanthato) iron(iii) (alkyl Et, Me, ^i^Pr and ^*n*^Pr) complexes. These complexes have been used for the deposition of iron sulfide, troilite (FeS) and pyrrhotite (Fe_1−*x*_S) nanostructured materials, using a simple, cheap and low temperature synthesis methods – spin coating and solventless pyrolysis. These provide control over nanocrystal size and morphologies by variation of temperature and precursor structure.^41,42^

## Experimental section

### Materials

Methanol (99.8%), 2-propanol (99.5%), *n*-propanol (99.9%), potassium ethyl xanthogenate (96%), carbon disulfide (low benzene 99.9%), chloroform (99.8%), and iron(iii) chloride (99%) were all acquired from Sigma-Aldrich, and used without further purification.

### Materials characterisation

Elemental analysis was conducted in the micro-analytical laboratory at the University of Manchester with a Carlo Erba EA 1108 elemental analyser. Thermogravimetric analysis (TGA) measurements were recorded from 0 °C to 600 °C at 10 °C min^−1^ heating rate under nitrogen, using a Seiko SSC/S200 TGA-DSC. Infrared spectra (IR) were obtained using a Specac single reflectance ATR instrument (4000–400 cm^−1^, resolution 4 cm^−1^). Melting points were obtained using a Barloworld SMP10 apparatus. Nuclear magnetic resonance (NMR) was recorded using a 400 MHz Bruker instrument. Mass spectra were recorded on Shimadzu Axima Confidence MALDI TOF mass spectrometer. Powder X-ray diffraction (p-XRD) measurements were carried out by using a Bruker D8 Advance and a Bruker Xpert diffractometer, utilising Cu-Kα radiation (1.54 Å). The samples were scanned between 20° and 80°, with a step size of 0.02° 2*θ*. Scanning electronic microscopy (SEM) images were obtained using a Philips XL30 FEG SEM. Inductively coupled plasma optical emission spectroscopy (ICP-OES) was carried out with a Perkin-Elmer Optima 5300 DV instrument. Raman spectra were recorded using a Renishaw 1000 microscope system equipped with laser excitation of 514 nm. Single crystal X-ray diffraction data for compounds were collected on a dual source Rigaku FR-X rotating anode diffractometer, using an MoK_α_ wavelength at 150 K, and reduced using a CrysAlisPro 171.39.30c.^43^ The structure was solved and refined using Shelx-2016, implemented through Olex2 v1.2.9.^44,45^

### Synthesis of molecular precursors

#### Synthesis of potassium methyl xanthate

Potassium methyl xanthate was synthesised according to a method in the literature.^46^ In a typical reaction, KOH (5 g, 89.12 mmol) was added to methanol (excess) and stirred. Thereafter, carbon disulfide (CS_2_) (6.78 g, 5.38 ml, 89.12 mmol) was added dropwise into the mixture. A yellow precipitate started to form, which was then filtered and recrystallised from acetone at room temperature and dried in the air to give potassium methylxanthate (KS_2_COMe) (12.2 g, 93.5%). Mp: 83–88 °C, elemental analysis for C_2_H_3_KOS_2_ (%): calc: C, 16.44, H, 2.07, S, 43.77; K, 26.76; found: C, 16.69; H, 2.01; S, 43.37; K, 27.13 FT-IR (*ν*_max_/cm^−1^): 2989 (w), 2932 (w), 1445 (w), 1428 (s), 1185 (s), 1085 (s), 1038 (s), 944 (w). ^1^H NMR (400 MHz, D_2_O): *δ* ppm 1.26 (s, 3H, CH_3_).

#### Synthesis of potassium isopropyl xanthate

Potassium isopropyl xanthate was synthesised by following the same method, using isopropanol (80 ml) (13.9 g 89.4%). Mp: 135–140 °C, elemental analysis for C_4_H_7_KOS_2_ (%): calc: C, 27.59, H, 4.05, S, 36.72; K, 22.45; found: C, 27.61.; H, 3.97; S, 36.53; K, 22.29. FT-IR (*ν*_max_/cm^−1^): 2969 (m), 1459 (w), 1382 (s), 1181 (s), 1127 (s), 1045 (s), 900 (s). ^1^H NMR (400 MHz, D_2_O): *δ* ppm 1.26 (d, 6H, CH_3_), 5.45 (m, 1H, CH).

#### Synthesis of potassium *n*-propyl xanthate

Potassium *n*-propyl xanthate was synthesised by following the same method, using *n*-propanol (80 ml) (13.7 g, 88.1%). Mp: 127–130 °C, elemental analysis for C_4_H_7_KOS_2_ (%): calc: C, 27.59, H, 4.05, S, 36.72; K, 22.45; found: C, 27.73.; H, 4.00; S, 36.73; K, 22.65. FT-IR (*ν*_max_/cm^−1^, powder): 2968 (s), 2936 (w), 2873 (w), 1452 (s), 1377 (w), 1270 (w), 1058 (s), 900 (s). ^1^H NMR (400 MHz, D_2_O): *δ* ppm 0.90 (t, 3H, CH_3_), 1.69 (m, 2H, CH_2_); 1.20 (t, 2H, CH_2_).

#### Synthesis of iron(iii) methylxanthate, Fe[S_2_COMe]_3_ (1)

An aqueous solution of (FeCl_3_) iron(iii) chloride (1.85 g, 11.41 mmol) (20 ml) was slowly added to a solution of ligand KS_2_COMe (5 g, 34.2 mmol) in distilled water (20 ml) while stirring. A black precipitate formed. The obtained solution was stirred constantly at room temperature for a further 10 min. The filtered black precipitate was washed with distilled water twice and dried under a vacuum overnight. The final product was a shiny black solid. Yield: (4.29 g 85.8%). Mp: 252–254 °C, elemental analysis: found: C, 19.24%; H, 3.07%; S, 50.56%; Fe, 13.56%; calc. C, 19.13%; H, 2.4%; S, 50.0%; Fe, 13.56%. IR (*ν*_max_/cm^−1^, powder): 2942 (w), 1436 (s), 1233 (s), 1161 (s), 1026 (s), 927 (s). ES^+^ (*m*/*z*): [M + H]^+^ 377.8.

#### Synthesis of iron(iii) ethylxanthate, Fe[S_2_COEt]_3_ (2)

Precursor (2) was synthesised by the same method of synthesis a precursor (1) using potassium ethylxanthate. The product formed was a black solid. Yield: (4.15 g 96.5%). Mp: 108–112 °C, elemental analysis: found: C, 25.15%; H, 3.54%; S, 44.46%; Fe, 13.01%; calc. C, 25.8%; H, 3.6%; S, 45.8%; Fe, 13.3%. IR (*ν*_max_/cm^−1^, powder): 2978 (w), 1743 (s), 1442 (w), 1366 (s), 1234 (s), 1107 (s), 997 (s). ES^+^ (*m*/*z*): [M + H]^+^ 418.8.

#### Synthesis of iron(iii) isopropylxanthate, Fe[S_2_CO^i^Pr]_3_ (3)

Precursor (3) was synthesised by the same method – of a precursor (1) using potassium iso-propyl xanthate. The final product was a shiny black solid precipitate. The complexes were recrystallized from chloroform at −20 °C. The yield was (3.8 g 86.1%). Mp: 83–88 °C, elemental analysis: Calc. C, 31.25; H, 4.59; S, 41.63; Fe, 12.12%; found: C, 30; 89; H, 4.54; S, 40.88; Fe, 11.73%. IR (*ν*_max_/cm^−1^, powder): 2976 (w), 1340 (s), 1081 (s), 1233 (s), 900 (s). ES^+^ (*m*/*z*): [M + H]^+^ 461.9.

#### Synthesis of iron(iii) *n*-propylxanthate, Fe[S_2_CO^*n*^Pr]_3_ (4)

Precursor (4) was synthesised by the same method as precursor (1), using potassium *n*-propyl xanthate. The final product was a shiny black solid precipitate. The complexes were recrystallized from chloroform at −20 °C. The yield was (4.1 g 92.9%). Mp: 75–80 °C, elemental analysis calc. C, 31.25; H, 4.59; S, 41.63; Fe, 12.12; found: C, 31.05; H, 4.52; S, 41.35; Fe, 11.87%. IR (*ν*_max_/cm^−1^): 2976 (w), 1340 (s), 1081 (s), 1233 (s), 900 (s). ES^+^ (*m*/*z*): [M + H]^+^ 461.9.

#### Deposition of thin films by spin coating

The deposition of iron sulfide thin films was carried out on glass substrates using precursors (1 to 4) and spin coating (Ossila, 24 V DC, 2.01 A). In a typical deposition process, 0.125 g of precursor was dissolved in 0.5 ml of chloroform. Then, 100 μL of precursor solution was deposited dropwise onto the glass substrate centre by the use of a micropipette. The rotation speed rate of the spin coater was set at 1400 rpm for 30 s. Once the process was complete, the coated substrates were left to dry for a few minutes at room temperature. Finally, the films were placed into a tube furnace and heated at different temperatures for 60 min, under an inert atmosphere.

#### Solid state pyrolysis of complexes

The solid precursor 0.4 g was placed in a ceramic boat and annealed in a tube furnace at a temperature, based on TGA decomposition data for 1 h under inert atmosphere.

## Results and discussion

The four tris(*O*-alkylxanthato) iron(iii) (alkyl = Me, Et, ^i^Pr and ^*n*^Pr) complexes, [Fe(S_2_COMe)_3_] (1), [Fe(S_2_COEt)_3_] (2), [Fe (S_2_CO^i^Pr)_3_] (3) and [Fe(S_2_CO^*n*^Pr)_3_] (4) were synthesised by metathesis of iron(iii) salts in water. All complexes were soluble in common organic solvents such as chloroform, THF and toluene. These complexes were stored at −20 °C to avoid premature decomposition.

### Single-crystal X-ray structure

**Fig. 1:**
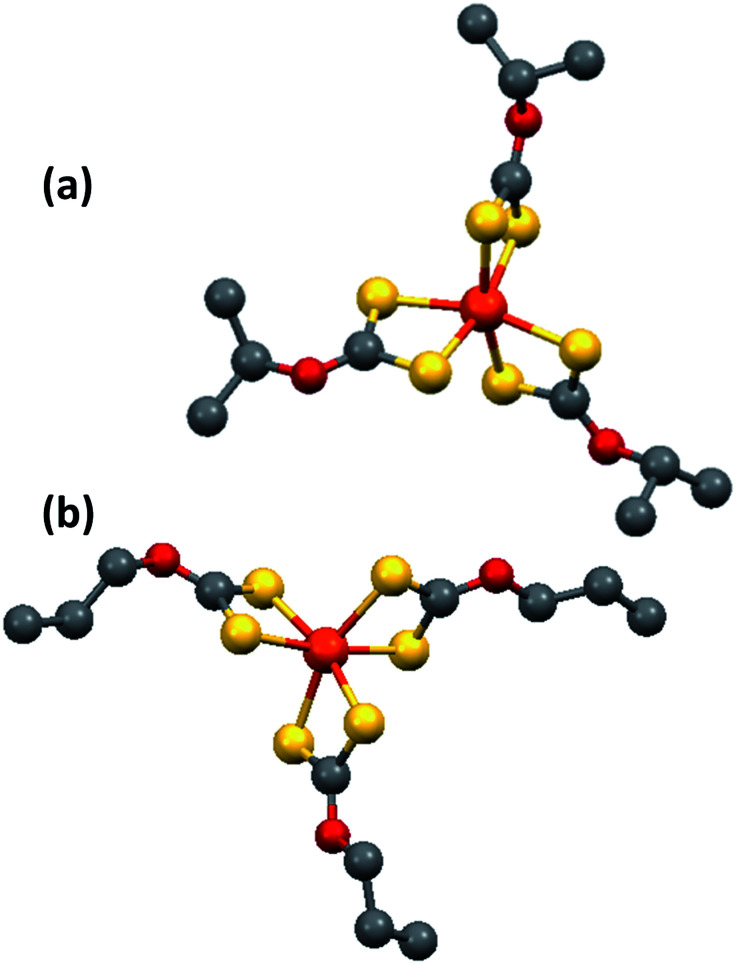
X-ray single crystal structure of (a) [Fe(S_2_CO^i^Pr)_3_] and (b) [Fe(S_2_CO^*n*^Pr)_3_]. Yellow spheres = sulfur, orange spheres = iron, red spheres = oxygen, grey spheres = carbon.

The single crystal X-ray structure of iron(iii) isopropylxanthate, Fe[S_2_CO^i^Pr]_3_ (3) and iron(iii) *n*-propylxanthate, Fe[S_2_CO^*n*^Pr]_3_ (4) are shown in Fig. 1. Complexes (3) and (4) crystallised in the monoclinic system with space group *P*2_1_/*c* and *P*2_1_/*n* respectively. The iron ion is coordinated to six sulfur atoms from the three bidentate xanthate ligands in a distorted octahedral environment.

The Fe–S bond length ranges in complex (3) are 2.29(8)–2.30(7) Å which is slightly longer than those for complex (4) which is in range of 2.2931–2.3162 Å. The Fe–S bond length obtained from both complexes is considerably closer to the distance of 2.3083–2.3263 Å reported previously for single crystal X-ray data of tris-(ethylxanthate) iron(iii).^47^ The refinement data is given in ESI Table S1.† Bond lengths and angles are reported in ESI Table S2 and S3.†

### Thermogravimetric analysis (TGA)

**Fig. 2:**
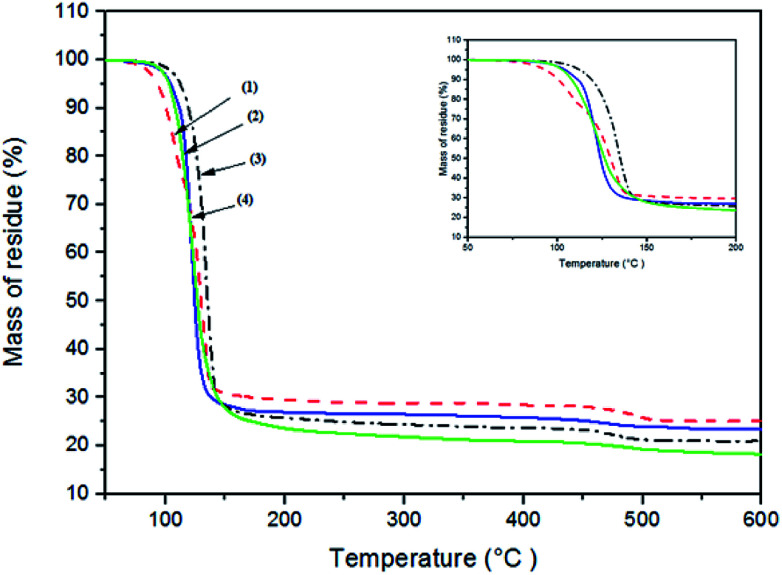
Thermogravimetric analysis (TGA) profiles of complexes [Fe(S_2_COMe)_3_] (1), [Fe(S_2_COEt)_3_] (2), [Fe(S_2_CO^i^Pr)_3_] (3), [Fe(S_2_CO^*n*^Pr)_3_] (4).

Thermal analysis of all complexes was performed up to 600 °C under a nitrogen atmosphere. The thermogram for complexes – [Fe(S_2_COEt)_3_] (2), [Fe(S_2_CO^i^Pr)_3_] (3) and [Fe(S_2_CO^*n*^Pr)_3_] (4) – show decomposition occurs through two steps, as shown in Fig. 2. These complexes exhibited approximately similar TGA profiles with a rapid mass loss within the temperature range of 120 to 300 °C, and gradual mass loss between 320 to 500 °C. [Fe(S_2_COMe)_3_] (1) decomposed in three steps, with the first step between 100 to 130 °C, and second and final steps similar to complexes (2–4). TGA results of the final residues are compiled in ESI Table S4.† All four complexes gave final solid residue amounts that matched with the calculated value for FeS_2_ or FeS. The complexes from (1) to (4) decomposed firstly to give FeS_2_; above 350 °C FeS is obtained from loss of sulfur.

### Deposition of thin films by spin coating

Iron sulfide precursors (1–4) were deposited on glass substrates by spin coating, using chloroform as solvent. Films of the precursors were then annealed at different growth temperatures, between 300 to 500 °C, in a furnace tube under N_2_ for 60 min. The resulting thin films of iron sulfides were uniform and black in colour and were characterised by p-XRD and SEM.

**Fig. 3:**
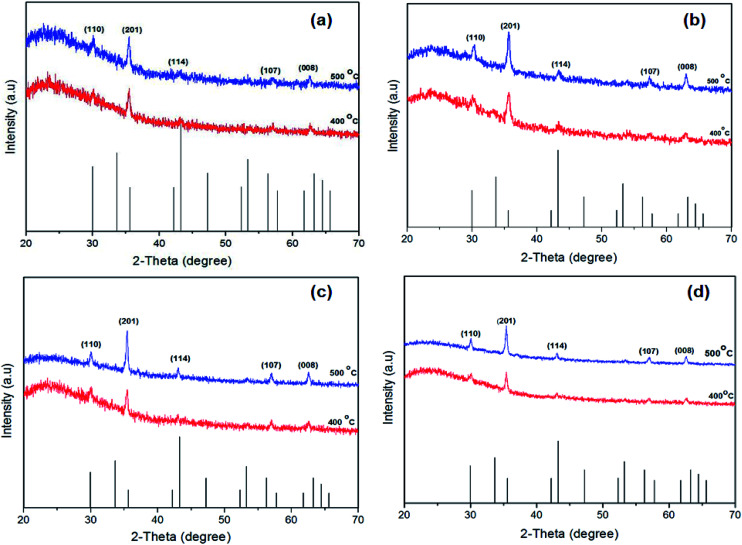
Powder X-ray diffraction (p-XRD) patterns of the iron sulfide thin films obtained by spin coating [Fe(S_2_COMe)_3_] (a), [Fe(S_2_COEt)_3_] (b), [Fe(S_2_CO^i^Pr)_3_] (c) and [Fe(S_2_CO^*n*^Pr)_3_] (d) followed by annealing under nitrogen at 400–500 °C for 60 min. The black sticks represent hexagonal troilite phase (FeS). (ICDD: 01-075-2165).

The p-XRD patterns of thin films prepared from a chloroform solution of [Fe(S_2_COMe)_3_], (1) [Fe(S_2_COEt)_3_] (2), [Fe(S_2_CO^i^Pr)_3_] (3) and [Fe(S_2_CO^*n*^Pr)_3_] (4) and annealed at three different temperatures (300, 400 and 500 °C) were recorded. The XRD pattern of iron sulfide thin films annealed at 400 and 500 °C corresponded to hexagonal troilite (FeS, ICDD no: 01-075-2165) Fig. 3. The diffraction peaks could be assigned to the (110), (201), (114), (107) and (008) planes. The p-XRD pattern of thin film of [Fe(S_2_COMe)_3_] (1) annealed at 300 °C exhibited a pure phase hexagonal troilite (FeS) (ESI Fig. S1†) No peaks were observed at a growth temperature below 400 °C when precursor [Fe(S_2_COEt)_3_] (2) was used. The p-XRD patterns of thin films deposited at 300 and 350 °C from a chloroform solution of [Fe(S_2_CO^i^Pr)_3_] (3) [Fe (S_2_CO^*n*^Pr)_3_] (4) show a similar trend to that observed from thin films deposited at 300 °C from complex (1) (ESI Fig. S2 and S3†). No crystalline deposition products were observed below 350 °C. It is clear that as annealing temperature increases that the peaks grow sharper as evidenced by a reduction of the full width at half maximum (FWHM); this generally indicates that the material becomes more microcrystalline according to the Scherrer equation.

Energy dispersive X-ray (EDX) spectroscopy of the iron sulfide thin films showed an approximately 1 : 1 ratio of Fe to S, consistent with troilite. The results are presented in ESI Table S5.†

**Fig. 4:**
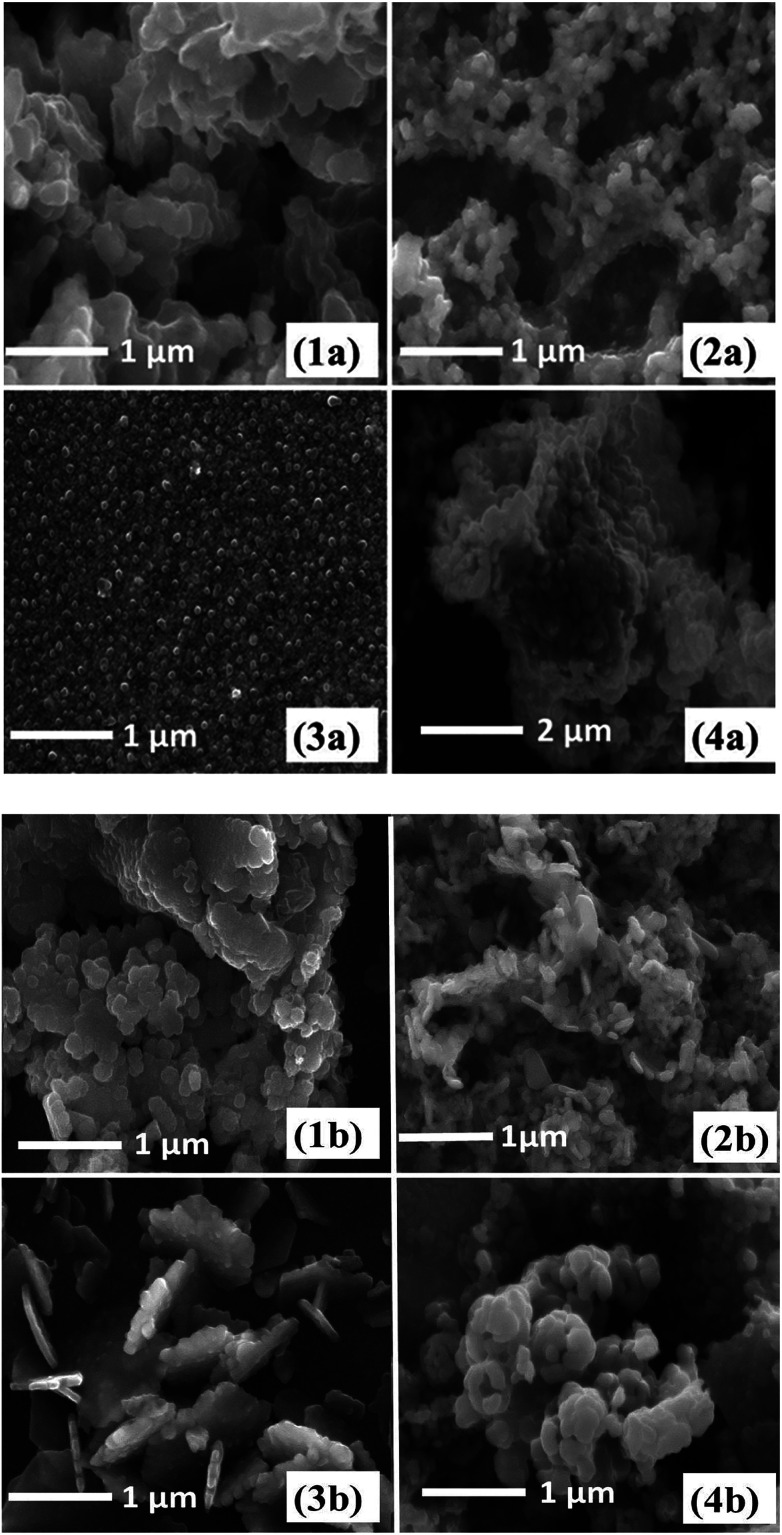
SEM images of iron sulfide nanoparticles by spin coating method from [Fe(S_2_COMe)_3_] (1a) at 400 °C and (1b) at 500 °C, [Fe(S_2_COEt)_3_] (2a) at 400 °C and (2b) at 500 °C, Fe(S_2_CO^i^Pr)_3_] (3a) at 400 °C and (3b) at 500 °C and [Fe(S_2_CO^*n*^Pr)_3_] (4a) at 400 °C and (4b) at 500 °C.

The surface morphology of iron sulfide thin films derived from pyrolysis of precursors (1–4) was studied using SEM. The SEM images obtained from an iron sulfide thin film derived from precursor (1) and deposited at 300 °C exhibited pseudo spherical crystallites in the size range of 260–300 nm (ESI Fig. S4†). At 400 and 500 °C clusters of crystallites with an average size of 200–250 nm (Fig. 4(1a) and (1b)) were apparent. The SEM images of iron sulfide thin films from [Fe(S_2_COEt)_3_] (2) at 400 and 500 °C are shown in Fig. 4(2a) and (2b) respectively. The morphology of the sample growth at 400 °C exhibited fairly uniform cluster like crystallites, consisting of spherical nanoparticles with open pores. At 500 °C, cluster-like crystallites consisting of irregular nanoparticles were obtained. SEM images of iron sulfide thin films derived from (3) at 300 °C show uniform cluster-like crystallites, as in ESI Fig. S5.† In contrast, the films deposited at 400 °C consist of smaller individual spherical crystallites with a size range of 140–180 nm, as in (Fig. 4(3a)). The films deposited at 500 °C show plate-like crystallites with an average size of 250–300 nm Fig. 3(3b). SEM images of iron sulfide thin films from (4) displayed the growth of randomly shaped crystallites at 350 °C (ESI Fig. S6†). The film growth at 400 °C showed a cluster of densely packed crystallites Fig. 4(4a), whereas a flower-like cluster were observed when films were deposited at 500 °C, as shown in Fig. 4(4b). In summary, the obtained images of the prepared thin films exhibited different sizes and morphologies. The SEM images of films deposited from all precursors show that the morphology of iron sulfide thin films is dependent on the growth temperature.

### Solid state thermolysis of complexes

**Fig. 5:**
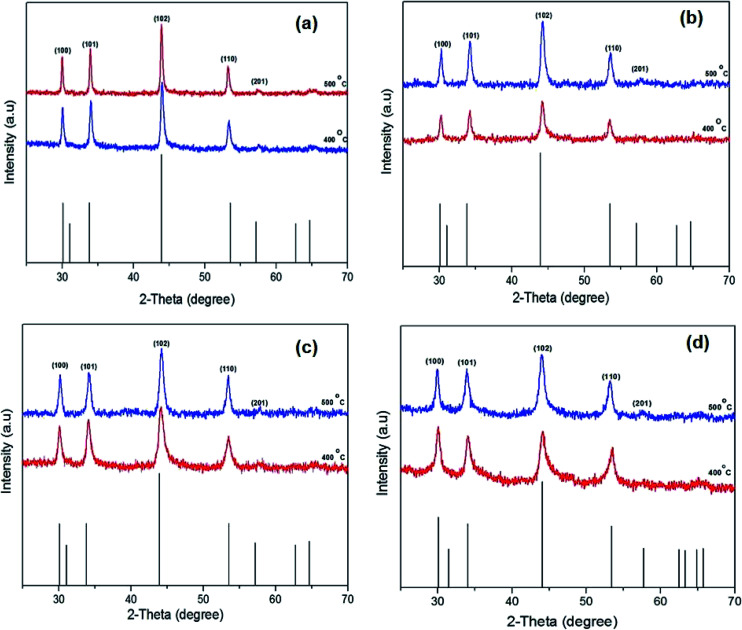
The powder X-ray diffraction (p-XRD) patterns of the iron sulfide nanostructure obtained by pyrolysis method [Fe(S_2_COMe)_3_] (a), [Fe(S_2_COEt)_3_] (b), [Fe(S_2_CO^i^Pr)_3_] (c) and [Fe(S_2_CO^*n*^Pr)_3_] (d) at temperature between 400 and 500 °C for 60 min. The black sticks represent hexagonal pyrrhotite (Fe_1−*x*_S) phase (ICDD no: 00-022-1120).

Solventless pyrolysis was employed for synthesis of iron sulfide crystallites using iron alkyl xanthates complexes (1–4). The solid complexes were decomposed under a nitrogen atmosphere in a tube furnace in the temperature range 300–500 °C for 60 min. The p-XRD patterns of the iron sulfide obtained from all four precursors [Fe(S_2_COMe)_3_] (1), [Fe(S_2_COEt)_3_] (2), [Fe(S_2_CO^i^Pr)_3_] (3) and [Fe(S_2_CO^*n*^Pr)_3_](4), at 400 and 500 °C as shown in Fig. 5(a)–(d). The results show the formation of a pure hexagonal pyrrhotite (Fe_1−*x*_S) phase (ICDD no: 00-022-1120). Reflections in the powder pattern corresponding to the (100), (101), (102), (110) and (201) planes of pyrrhotite (Fe_1−*x*_S) were dominant in the sample obtained from all precursors. The XRD patterns obtained from the complexes [Fe(S_2_CO^i^Pr)_3_] (3) and [Fe(S_2_CO^*n*^Pr)_3_] (4) gave a pure pyrrhotite (Fe_1−*x*_S) at all growth temperatures of 300, 400 and 500 °C, while [Fe(S_2_COMe)_3_] (1) and [Fe(S_2_COEt)_3_], (2) gave a pure phase at high temperatures of 400 °C and 500 °C. However, the XRD patterns obtained at a growth temperature of 300 and 350 °C from the complex [Fe(S_2_COMe)_3_] (1) and [Fe(S_2_COEt)_3_] (2), respectively show that some additional peaks corresponded to cubic pyrite (FeS_2_) (ICDD no: 01-071-0053) (denoted by the symbol*) (ESI Fig. S7 and S8†). The average crystallites size of iron sulfide is affected by growth temperature. The average crystallite size of iron sulfide from precursor (1) to (4) at two different temperatures 400 and 500 °C were calculated using the Scherer equation (ESI Table S6†). The average crystallite size of iron sulfide from complex (1) at growth temperature of 400 °C was 22.3 ± 2.6 nm. Whereas the crystalline obtained from growth temperature of 500 °C has an estimated diameter of 28.6 ± 1.6 nm the observed decrement in crystalline size with temperature indicated the influence of temperature. ICP-OES and EDX spectroscopy of iron sulfide samples confirmed the phase obtained from samples at high temperature of 500 °C is pyrrhotite Fe_1−*x*_S summary of results are shown in (ESI Table S7 and S8†). In addition, Raman spectroscopy confirmed the formation of pyrrhotite Fe_1−*x*_S with three vibration mode observed at wavenumbers of 214, 284 cm^−1^ and 399 cm^−1^ for all complexes (1–4).

**Fig. 6:**
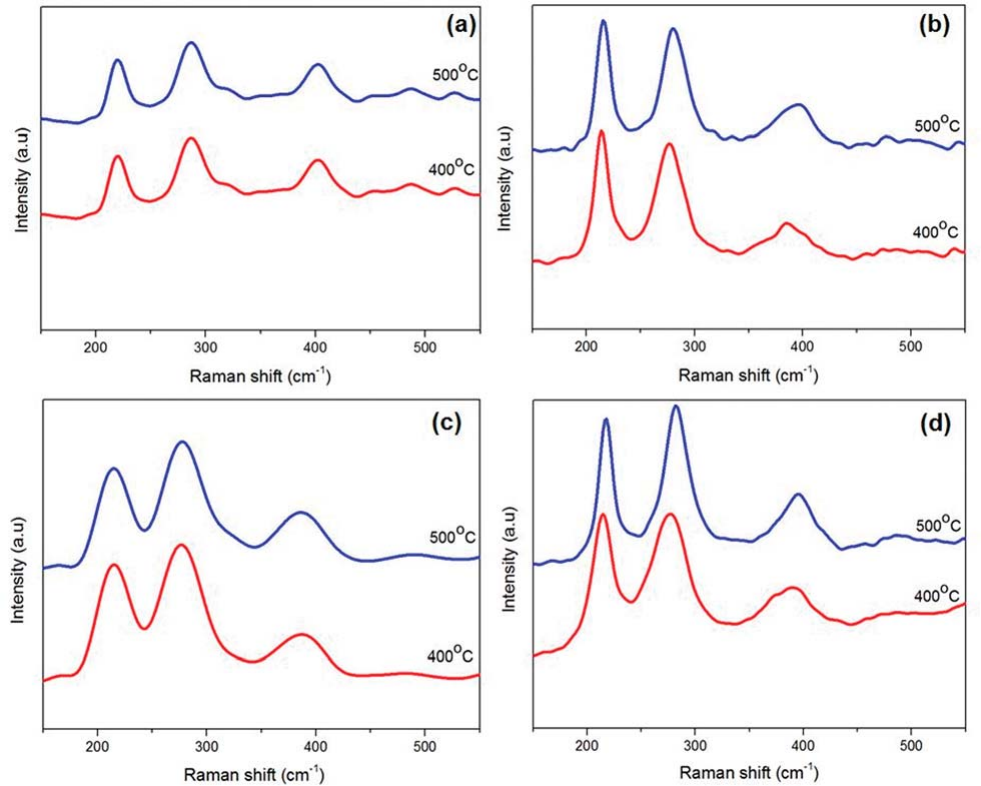
Raman spectra of hexagonal pyrrhotite phase (Fe_1−*x*_S) from complexes [Fe(S_2_COMe)_3_] (a), [Fe(S_2_COEt)_3_] (b), [Fe(S_2_CO^i^Pr)_3_] (c) and[Fe(S_2_CO^*n*^Pr)_3_] (d) at 400 and 500 °C.

Our results are in good agreement with that reported previously for iron sulfide pyrrhotite.^48–50^ The Raman spectra of pyrrhotite obtained from the pyrolysis of complexes (1–4) are presented in Fig. 6 and ESI Fig. S11.†

**Fig. 7:**
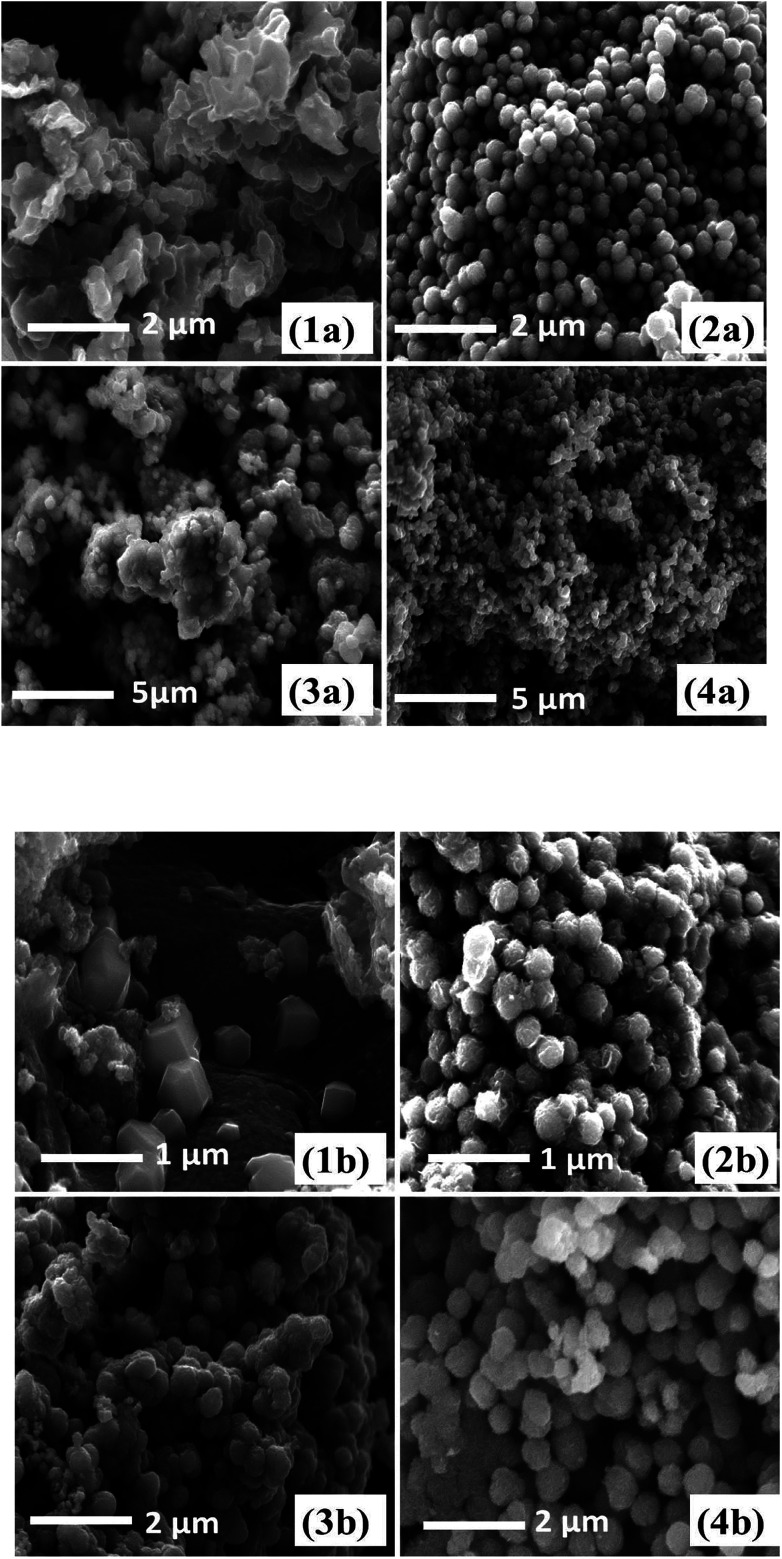
SEM images of iron sulfide nanoparticles from solventless pyrolysis of [Fe(S_2_COMe)_3_] (1a) at 400 °C and (1b) at 500 °C, [Fe(S_2_COEt)_3_] (2a) at 400 °C and (2b) at 500. [Fe(S_2_CO^i^Pr)_3_] (3a) at 400 °C and (3b) at 500 °C and [Fe(S_2_CO^*n*^Pr)_3_] (4a) at 400 °C and (4b) at 500 °C.

SEM images of the iron sulfide crystallites growth at 400 °C and 500 °C derived from the thermolysis of complex (1) are presented in Fig. 7. The morphology of crystallites at growth temperatures of 300 °C (ESI Fig. S12†) and 400 °C showed clusters of densely packed crystallites, whilst hexagonal crystallites were observed at a growth temperature of 500 °C. SEM images from the pyrolysis of precursor [Fe(S_2_COEt)_3_] (2) at 300 °C (ESI Fig. S13†) and 400 and 500 °C are shown in Fig. 7. At all growth temperatures of 300–500 °C, regular spherical-like crystallites were observed. SEM images of iron sulfide crystals grown from the pyrolysis of [Fe(S_2_CO^i^Pr)_3_] (3) and [Fe(S_2_CO^*n*^Pr)_3_] (4) exhibited similar morphologies with spherical-like crystals observed and with considerable variation in particle size as shown in Fig. 7. The morphology of crystallites obtained from complexes (1–4) was clearly dependent on the temperature and the type of precursor.

## Conclusion

In summary, a series of iron alkyl xanthate complexes, including [Fe(S_2_COMe)_3_] (1) [Fe(S_2_COEt)_3_] (2) [Fe(S_2_CO^i^Pr)_3_] (3) and [Fe(S_2_CO^*n*^Pr)_3_] (4) have been successfully synthesised, using a single-source route. The X-ray crystal structures of [Fe(S_2_CO^i^Pr)_3_] (3) and [Fe(S_2_CO^*n*^Pr)_3_] (4) have been determined. These four complexes were used for the deposition of iron sulfide crystallites. Two simple methods have been described for the growth of iron sulfide nanocrystals which are spin coating and solventless pyrolysis methods. Different deposition parameters such as deposition method, deposition temperature and precursor type were investigated in this study. The deposited iron sulfide phases and the morphology of iron sulfide crystallites were significantly influenced by the deposition method used. p-XRD results revealed the formation of troilite when a spin coat-annealing method was used while iron sulfide pyrrhotite Fe_1−*x*_S was mainly formed when the solventless pyrolysis method was used. Both methods are promising for the low temperature production of iron sulfide materials with control of the crystalline phase produced.

## Conflicts of interest

There are no conflicts of interest to declare.

## Supplementary Material

RA-008-C8RA04917C-s001

RA-008-C8RA04917C-s002
