# Supplementary material for: Synthesis of nanostructured powders and thin films of iron sulfide from molecular precursors
Source: RSC Adv. 2018 Aug 15;8(51):29096–103. doi: 10.1039/c8ra04917c (PMC9084487; doi:10.1039/c8ra04917c)
Supplement: RA-008-C8RA04917C-s001 [file RA-008-C8RA04917C-s001.pdf]

Electronic Supplementary Material (ESI) for RSC Advances.
This journal is © The Royal Society of Chemistry 2018

Synthesis of Nanostructured Powders and Thin Films of Iron Sulfide from Molecular Precursors

Laila Almanqur,[a] Inigo Vitorica-yrezabal ,George Whitehead[a] David J. Lewis[b]* and Paul O'Brien[ab]*

[a]School of Chemistry

[b]School of Materials

The University of Manchester

Oxford Road, Manchester, UK M13 9PL

Electronic Supporting Information

Table S1. Crystallographic data for of $[Fe(S_2CO^iPr)_3]$ **(3)** and $[Fe(S_2CO^nPr)_3]$ **(4)**

**Compound**	**(3)**	**(4)**
**Empirical formula**	$C_{12}H_{21}FeO_3S_6$	$C_{12}H_{21}FeO_3S_6$
**Formula weight**	461.50	461.50
**Temperature/K**	150.00(10)	150.00(2)
**Crystal system**	monoclinic	monoclinic
**Space group**	$P2_1/n$	$P2_1/c$
**a/Å**	9.8787(2)	13.9928(3)
**b/Å**	9.6899(2)	15.6007(3)
**c/Å**	21.8298(5)	9.13185(19)
**α/°**	90	90
**β/°**	101.469(2)	102.292(2)
**γ/°**	90	90
**Volume/Å^3**	2047.90(8)	1947.76(7)
**Z**	4	4
**ρ_{calc}g/cm^3**	1.497	1.574
**μ/mm^{-1}**	1.354	1.423
**F(000)**	956.0	956.0
**Crystal size/mm^3**	0.4 × 0.35 × 0.2	0.522 × 0.204 × 0.094
**Radiation**	MoKα (λ = 0.71073)	MoKα (λ = 0.71073)
**2Θ range for data collection/°**	4.26 to 59.988	3.962 to 52.736
**Index ranges**	$-13 \le h \le 13$, $-13 \le k \le 12$, $-29 \le l \le 27$	$-17 \le h \le 17$, $-19 \le k \le 19$, $-11 \le l \le 10$
**Reflections collected**	36718	27884
**Independent reflections**	5332 [R_{int} = 0.0282, R_{sigma} = 0.0191]	3978 [R_{int} = 0.0217, R_{sigma} = 0.0130]
**Data/restraints/parameters**	5332/0/205	3978/0/202
**Goodness-of-fit on F^2**	1.029	1.035
**Final R indexes [I>=2σ (I)]**	R_1 = 0.0231, wR_2 = 0.0517	R_1 = 0.0177, wR_2 = 0.0439
**Final R indexes [all data]**	R_1 = 0.0288, wR_2 = 0.0537	R_1 = 0.0194, wR_2 = 0.0446
**Largest diff. peak/hole / e Å^{-3}**	0.43/-0.40	0.35/-0.22

.

Table S2. Bond length and angles for complex **(3)**

**Bond length and angle**	**Data**
Fe1- S7	2.2958(4)
Fe1- S14	2.3074(4)
Fe1- S21	2.2846(4)
Fe1- S2	2.3177(4)
Fe1- S16	2.2972(4)
Fe1- S9	2.2981(4)
S7- C3	1.6907(13)
S14- C10	1.6846(13)
S21- C17	1.6922(14)
S2- C3	1.6937(13)
S16- C17	1.6954(14)
S9- C10	1.6954(14)
S7- Fe1- S14	95.195(14)
S14- Fe1- S2	96.812(14)
S16- Fe1- S9	92.051(14)

Table S3. Bond length and angles for complex **(4)**

**Bond length and angle**	**Data**
Fe1- S6	2.3013(3)
Fe1- S2	2.3155(4)
Fe1- S1	2.2948(4)
Fe1- S5	2.2931(4)
Fe1- S3	2.3161(4)
Fe1- S4	2.3050(4)
S6- C9	1.6908(13)
S2- C1	1.6934(12)
S1- C1	1.6851(13)
S5- C9	1.6971(13)
S3- C5	1.6888(13)
S4- C5	1.6851(13)
S6- Fe1- S2	99.464(13)
S2- Fe1- S3	159.345(14)
S1- Fe1- S6	170.879(14)

Table S4. Decomposition data of precursors[$Fe(S_2COMe)_3$] **(1)**, [$Fe(S_2COEt)_3$] **(2)**, [$Fe(S_2CO^iPr)_3$] **(3)** and [$Fe(S_2CO^nPr)_3$]**(4).**

	Complex	Iron sulfide Phase	Mass loss% Calculated%	Found%	Decomposition temperature °C
**1**	[$Fe(S_2COMe)_3$]	FeS_2	31.8%	30.2%	250-350 °C
		FeS	23.3%	24.5%	500-550 °C
**2**	[$Fe(S_2COEt)_3$]	FeS_2	28.6%	28.3%	290-350 °C
		FeS	20.9%	21.2%	500-550 °C
**3**	[$Fe(S_2CO^iPr)_3$]	FeS_2	26%	26.9%	270-300 °C
		FeS	19.1%	20%	400-450 °C
**4**	[$Fe(S_2CO^nPr)_3$]	FeS_2	26%	25.1%	200-250 °C
		FeS	19.1%	18.8%	500-550 °C

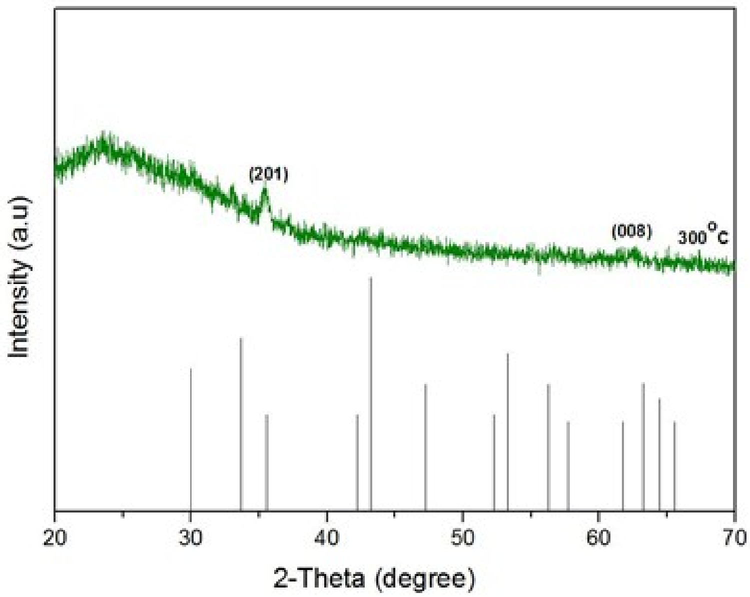

Figure S1. The XRD patterns of iron sulfide thin films prepared by spin coating method from [Fe ($S_2COMe)_3$] complex heated at 300°C for 60 min. The black sticks represent hexagonal troilite phase (FeS). (ICDD: 01-075-2165).

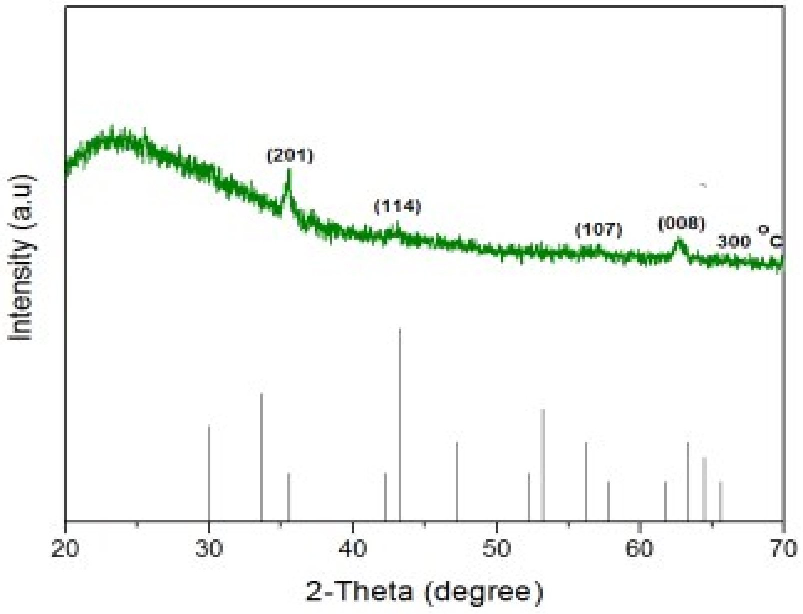

Figure S2. The XRD patterns of iron sulfide thin films prepared by spin coating method from [Fe $(S_2CO^iPr)_3$] complex heated at 300 °C for 60 min. The black sticks represent hexagonal troilite phase (FeS). (ICDD: 01-075-2165).

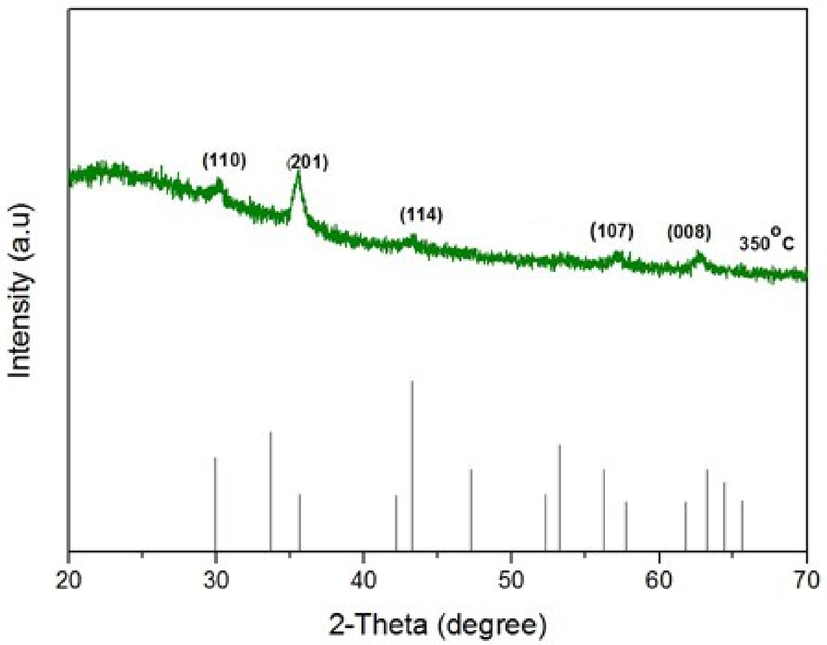

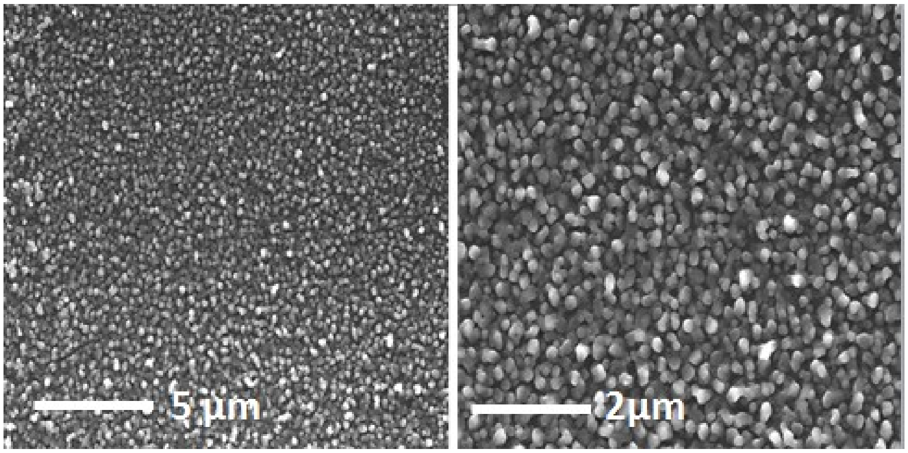

FigureS4. SEM images of iron sulfide thin films from complex [$Fe(S_2COMe)_3$] deposited by the spin coating method at 30O ◦C

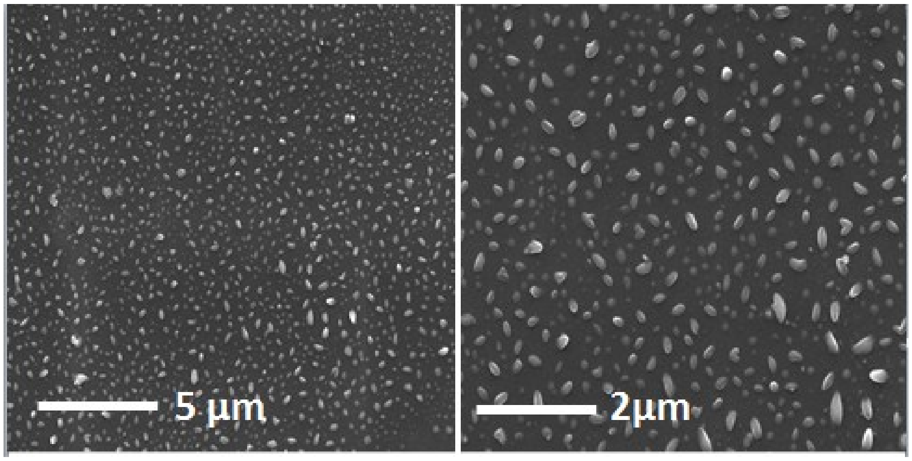

Figure S5. SEM images of iron sulfide thin films from complex [$Fe(S_2CO^ipr)_3$] deposited by the spin coating method at 300 ◦C

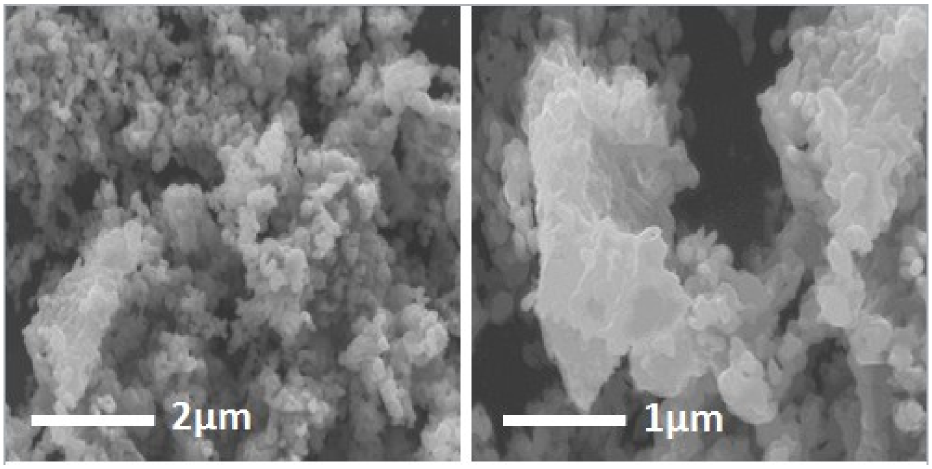

Figure S6. SEM images of iron sulfide thin films from complex $[Fe(S_2CO^npr)_3]$ deposited by the spin coating method at 350 °C

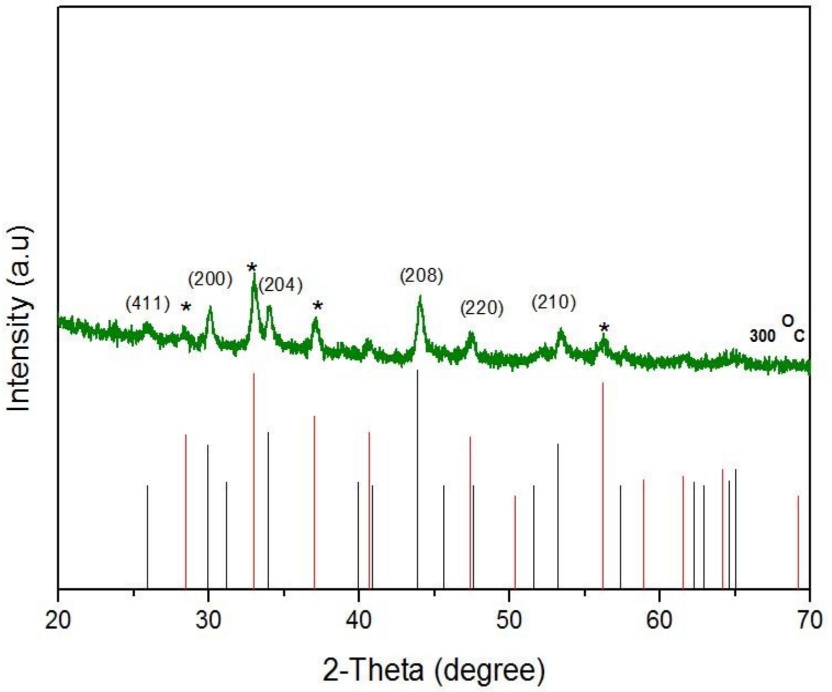

Figure S7. The XRD patterns of iron sulfide nanoparticles prepared by melt method from [Fe $(S_2COMe)_3$] complex heated at 300 °C for 60 min. The black sticks represent hexagonal pyrrhotite phase ($Fe_{1-x}S$). The red sticks represent pyrite FeS_2 phase (denoted by symbol (*)).

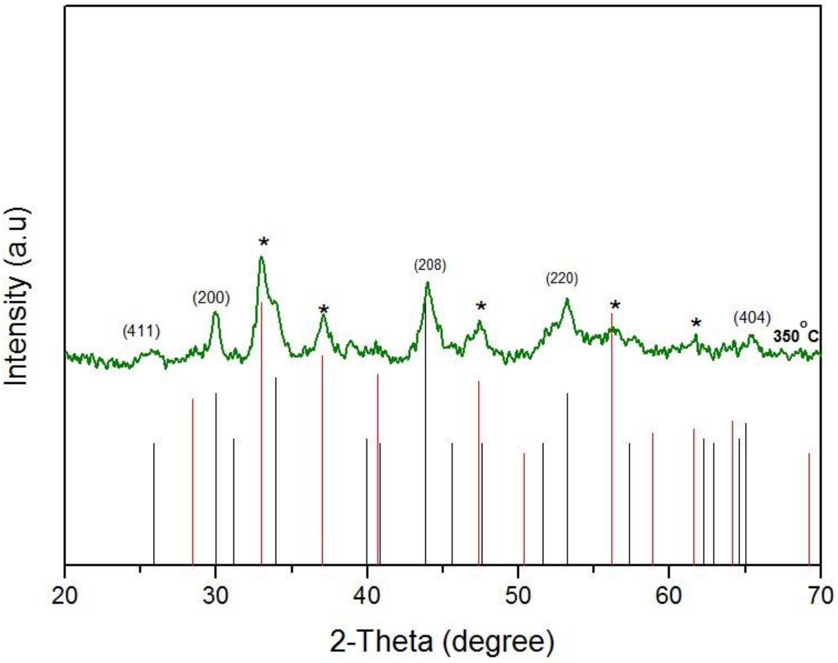

Figure S8. The XRD patterns of iron sulfide thin films prepared by melt method from [Fe $(S_2COEt)_3$] complex heated at 350 °C for 60 min. The black sticks represent hexagonal pyrrhotite phase ($Fe_{1-x}S$). The red sticks represent pyrite FeS_2 phase (denoted by symbol (*).

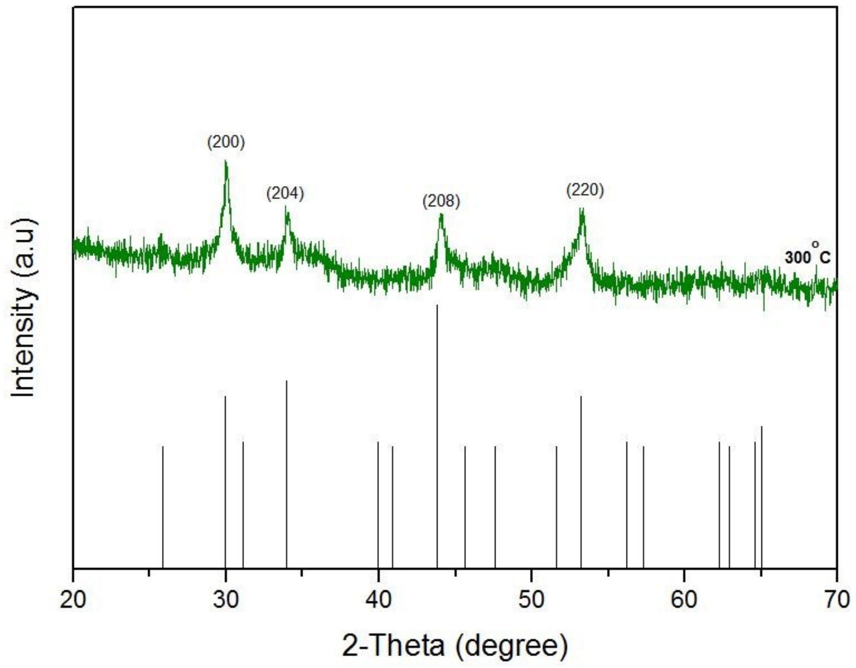

Figure S9. The XRD patterns of iron sulfide nanoparticles prepared by melt method from [Fe ($S_2CO^iPr)_3$] complex heated at 300 °C for 60 min. The black sticks represent hexagonal pyrrhotite phase ($Fe_{1-x}S$).

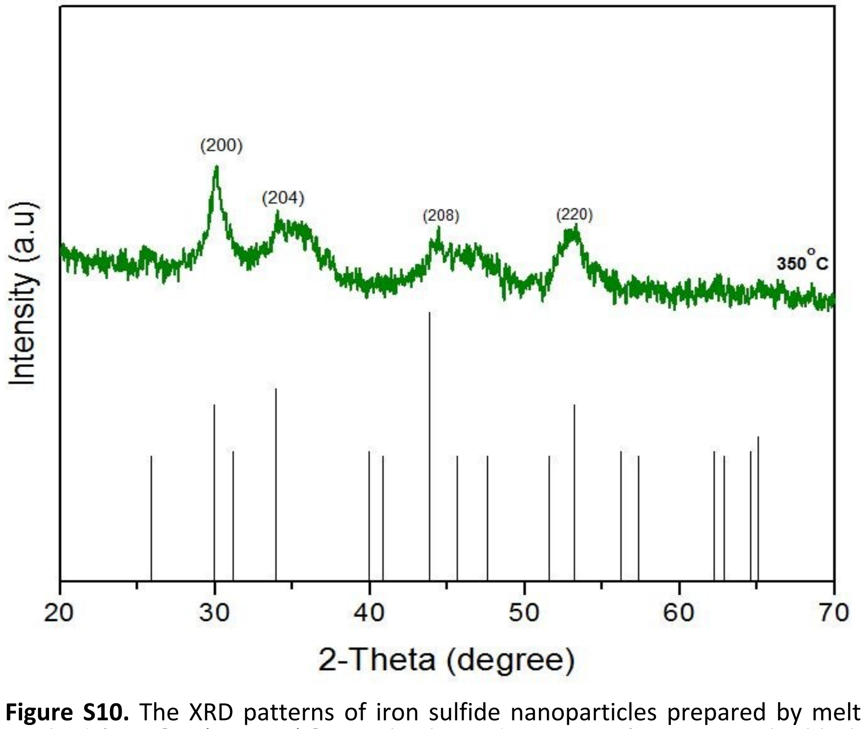

Figure S10. The XRD patterns of iron sulfide nanoparticles prepared by melt method from [Fe ($S_2CO^nPr)_3$] complex heated at 350 °C for 60 min. The black sticks represent hexagonal pyrrhotite phase ($Fe_{1-x}S$).

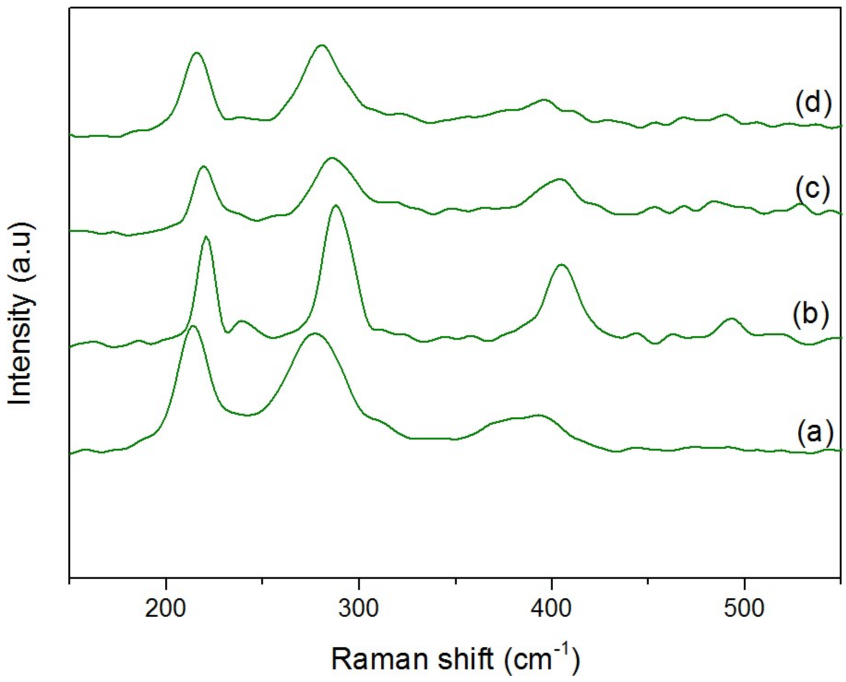

Figure S11. Raman spectra of hexagonal pyrrhotite phase ($Fe_{1-x}S$). from complexes $[Fe(S_2COMe)_3]$ **(a)** at 300 °C , $[Fe(S_2COEt)_3]$ **(b)** at at 350 °C , $[Fe(S_2CO^iPr)_3]$ **(c)** at 300 °C and$[Fe(S_2CO^nPr)_3]$**(d)** at 350 °C.

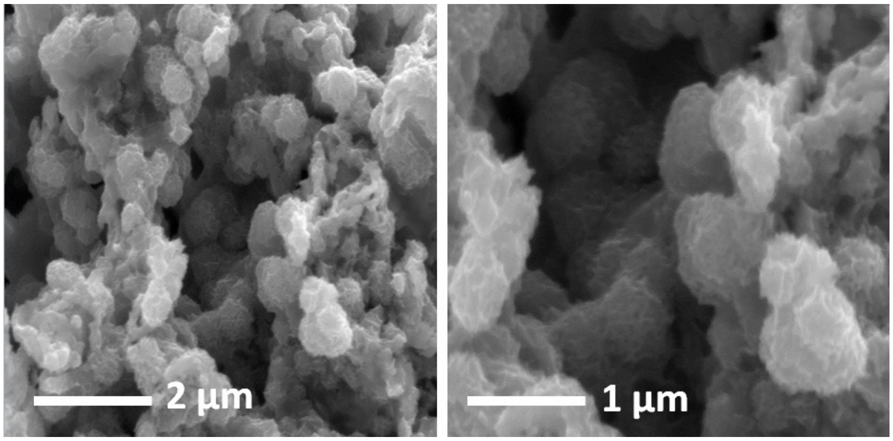

Figure S12. SEM images of iron sulfide nanoparticles from complex $[Fe(S_2COMe)_3]$ by melt method at 300 °C

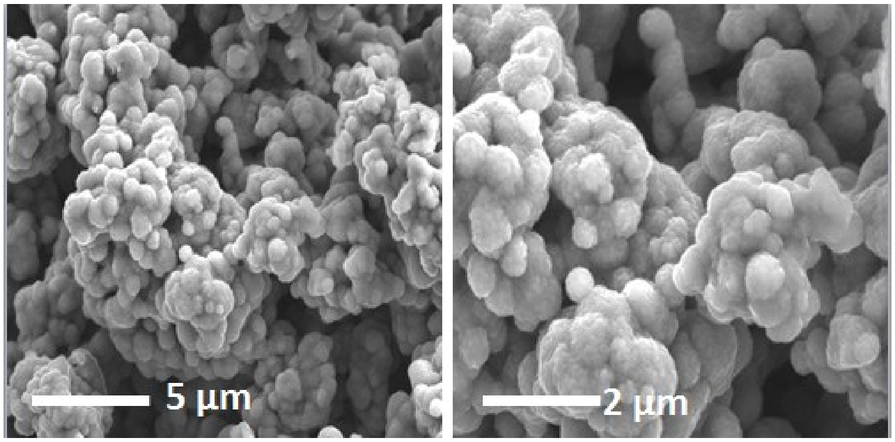

Figure S13. SEM images of iron sulfide nanoparticals from complex [Fe(S_2COEt)$_3$] by melt method at 350 °C

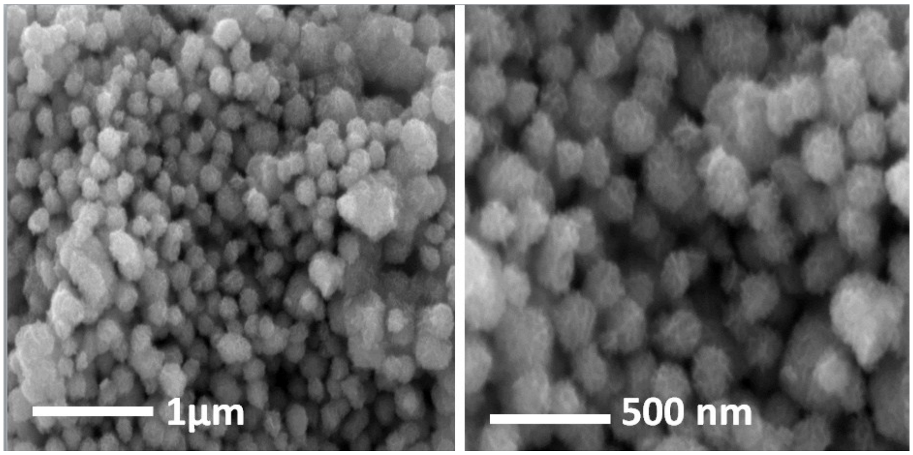

Figure S14. SEM images of iron sulfide nanoparticles from complex [Fe(S_2CO^{i}Pr)$_3$] by melt method at 350 °C

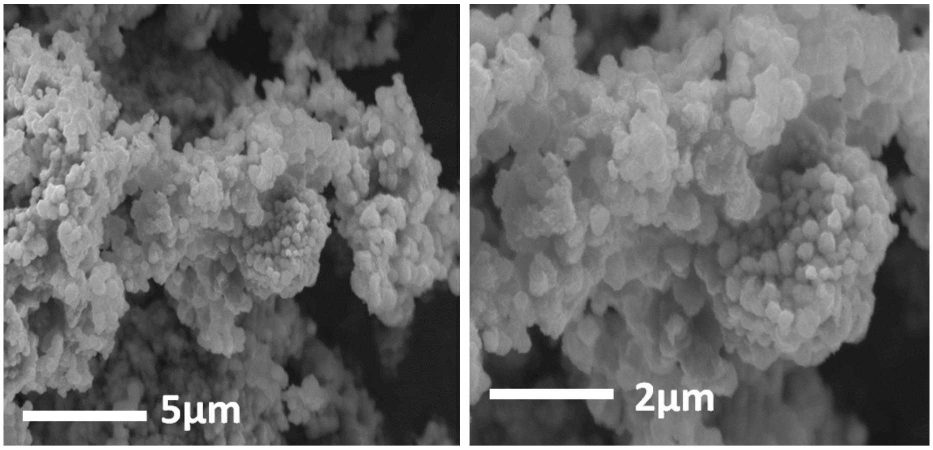

Figure S15. SEM images of iron sulfide nanoparticles from complex [Fe(S_2CO^{n}Pr)$_3$] using melt method at 300 °C

Table S5. Elemental composition of Fe and S in iron sulfide thin films heated at 500 °C found by EDX.

Complex	Fe (%)	S (%)	Ratio (Fe:S)
**[Fe(S_2COMe)$_3$] (1)**	49.7	50.0	FeS
**[Fe(S_2COEt)$_3$] (2)**	49.3	50.6	FeS
**[Fe(S_2CO^{i}Pr)$_3$] (3)**	49.9	50.0	FeS
**[Fe(S_2CO^{n}Pr)$_3$] (4)**	49.4	50.1	FeS

The average crystallite size of iron sulfide from precursor **(1)** to **(4)** at two different temperatures 400 and 500 °C were calculated using the Schererr equation:

$L = K\lambda / B \cos(\theta)$

Where L, K, λ, B and θ are crystallite size, 0.94, the X-ray wavelength, the full width at half maximum of the peak and Bragg diffraction angle respectively.[1]

Table S6. The average crystallite size of nanocrystals from complex **(1)**, **(2)**, **(3)** and **(4)** at growth temperature of 400 °C and 500 °C

**Precursor**	**Average crystallite size at 400 °C**	**Average crystallite size at 500 °C**
**[Fe(S_2COMe)$_3$] (1)**	22.3 nm	28.6 nm
**[Fe(S_2COEt)$_3$] (2)**	14. 2 nm	17.7 nm
**[Fe(S_2CO^{i}Pr)$_3$] (3)**	7.6 nm	10.2 nm
**[Fe(S_2CO^{n}Pr)$_3$] (4)**	11.2 nm	14 nm

Table S7. Elemental composition of Fe and S in iron sulfide samples heated at 500 °C found by ICP-OES.

Complex	Fe (%)	S (%)	Ratio (Fe:S)
**$[Fe(S_2COMe)_3]$ (1)**	44.0	55.9	$Fe_{0.78}$ S
**$[Fe(S_2COEt)_3]$ (2)**	44.3	55.6	$Fe_{0.79}$ S
**$[Fe(S_2CO^iPr)_3]$ (3)**	45.3	54.6	$Fe_{0.82}$ S
**$[Fe(S_2CO^nPr)_3]$ (4)**	44.0	55.7	$Fe_{0.79}$ S

Table S8. Elemental composition of Fe and S in iron sulfide samples heated at 500 °C found by EDX.

Complex	Fe (%)	S (%)	Ratio (Fe:S)
**$[Fe(S_2COMe)_3]$ (1)**	44.0	55.8	$Fe_{0.78}$ S
**$[Fe(S_2COEt)_3]$ (2)**	44.2	55.6	$Fe_{0.79}$ S
**$[Fe(S_2CO^iPr)_3]$ (3)**	45.1	54.3	$Fe_{0.83}$ S
**$[Fe(S_2CO^nPr)_3]$ (4)**	44.2	55.9	$Fe_{0.79}$ S

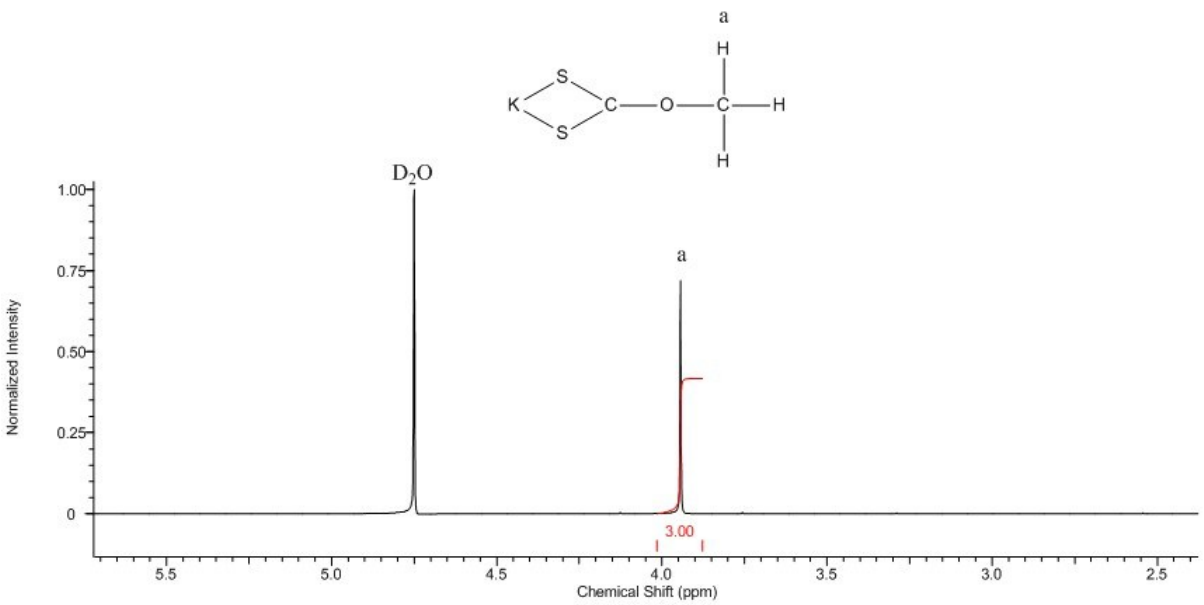

Figure S16. ^{1}H NMR spectra of iron (III) methylxanthate, $[Fe(S_2COMe)]_3$**(1)**

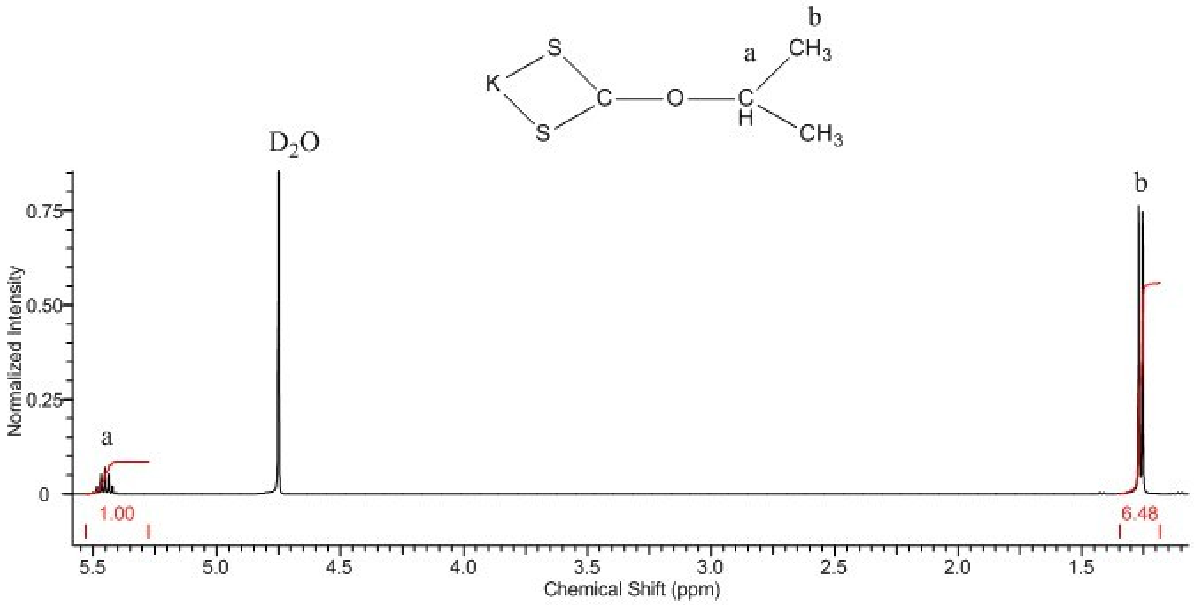

Figure S17. ^{1}H NMR spectra of iron (III) isopropylxanthate $[Fe(S_2CO^iPr)_3]$**(3)**

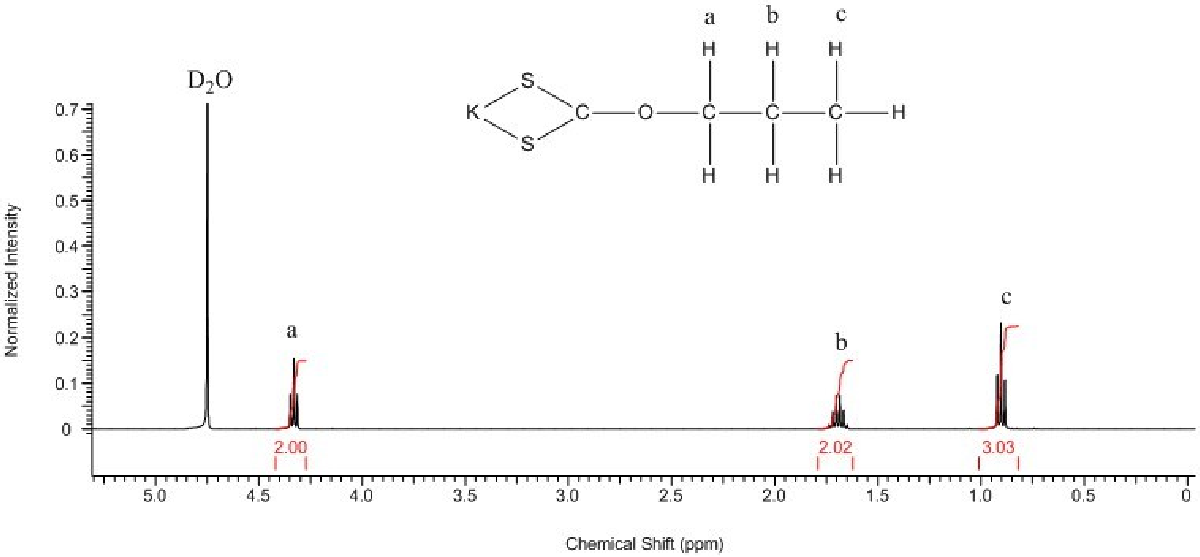

Figure S18. ^{1}H NMR spectra of iron (III) n-propylxanthate $[Fe(S_2CO^nPr)]_3$**(4)**

References

1. L. Pallon, R. Olsson, D. Liu, A. Pourrahimi, M. Hedenqvist, A. Hoang, S. Gubanski and U. Gedde, *J. Mater. Chem. A*, 2015, **3**, 7523-7534.
